# A microbially produced AhR ligand promotes a Tph1-driven tolerogenic program in multiple sclerosis

**DOI:** 10.1038/s41598-024-57400-8

**Published:** 2024-03-20

**Authors:** Teresa Zelante, Giuseppe Paolicelli, Francesca Fallarino, Marco Gargaro, Gianluca Vascelli, Marco De Zuani, Jan Fric, Petra Laznickova, Marcela Hortova Kohoutkova, Antonio Macchiarulo, Daniela Dolciami, Giuseppe Pieraccini, Lorenzo Gaetani, Giulia Scalisi, Caterina Trevisan, Barbara Frossi, Carlo Pucillo, Antonella De Luca, Emilia Nunzi, Roberta Spaccapelo, Marilena Pariano, Monica Borghi, Francesca Boscaro, Riccardo Romoli, Andrea Mancini, Lucia Gentili, Giorgia Renga, Claudio Costantini, Matteo Puccetti, Stefano Giovagnoli, Maurizio Ricci, Martina Antonini, Paolo Calabresi, Paolo Puccetti, Massimiliano Di Filippo, Luigina Romani

**Affiliations:** 1 Department of Medicine and Surgery, University of Perugia, Piazza Lucio Severi, 1, 06132 Perugia, Italy; 2 International Clinical Research Centre, St. Anne’s University Hospital Brno, Brno, Czech Republic; 3 Institute of Hematology and Blood Transfusion, U Nemocnice 2094/1, 128 20 Prague, Czech Republic; 4 International Clinical Research Centre, Faculty of Medicine, Masaryk University, Kamenice 5, 625 00 Brno, Czech Republic; 5 Department of Pharmaceutical Science, University of Perugia, 06132 Perugia, Italy; 6 Mass Spectrometry Center (CISM), University of Florence, 50139 Florence, Italy; 7 Department of Medical and Biological Science, University of Udine, 33100 Udine, Italy; 8 Center of Functional Genomics, C.U.R.Ge.F, University of Perugia, 06132 Perugia, Italy; 9 Unità di Neurologia, Fondazione Policlinico Universitario Agostino Gemelli, IRCCS, Rome, Italy; 10 Interuniversity Consortium for Biotechnology, (CIB), 34149 Trieste, Italy

**Keywords:** Mast cells, Aryl hydrocarbon receptor, Serotonin, 3-IAld, Multiple sclerosis, Autoimmunity, Immunology, Pathogenesis

## Abstract

Multiple sclerosis is a debilitating autoimmune disease, characterized by chronic inflammation of the central nervous system. While the significance of the gut microbiome on multiple sclerosis pathogenesis is established, the underlining mechanisms are unknown. We found that serum levels of the microbial postbiotic tryptophan metabolite indole-3-carboxaldehyde (3-IAld) inversely correlated with disease duration in multiple sclerosis patients. Much like the host-derived tryptophan derivative l-Kynurenine, 3-IAld would bind and activate the Aryl hydrocarbon Receptor (AhR), which, in turn, controls endogenous tryptophan catabolic pathways. As a result, in peripheral lymph nodes, microbial 3-IAld, affected mast-cell tryptophan metabolism, forcing mast cells to produce serotonin via Tph1. We thus propose a protective role for AhR–mast-cell activation driven by the microbiome, whereby natural metabolites or postbiotics will have a physiological role in immune homeostasis and may act as therapeutic targets in autoimmune diseases.

## Introduction

Commensal microorganisms of the microbiota are able to release diverse metabolites or small molecules with different targets, modulating immunity and several physiological functions. Numerous molecules act as anti-bacterial to remove pathogenic organisms^1^. Remarkably, gut bacterial species transform dietary amino acids into diverse end-products^2,3^. For instance, the indole-3-pyruvic acid (IPyA) route is fundamental to convert aromatic amino acids to aroma compounds via transamination of tryptophan (Trp)^4^. IPyA secondary metabolites include indol-3-acetaldehyde (3-IAAld), indole-3-acetic-acid (IAA) and indole-3-carboxaldehyde (3-IAld)—all known as being Aryl hydrocarbon Receptor (AhR) ligands^3,5,6^.

The AhR is activated by small molecules provided by the diet, microorganisms, metabolism, and pollutants^7^ and thus constitutes a potential target for therapeutic immuno-modulation^8^. Because AhR ligands are known immuno-modulators in multiple sclerosis (MS)^8–10^, in particular by diet administration^11^, here we have investigated the impact of systemic administration of 3-IAld in a mouse model of MS, thus proposing a protective role for AhR activation driven by the microbiome. As described in demyelinated multiple sclerosis plaques, mast cells play an important role in autoimmune disease and represent a route for infiltrating cells to enter the brain in inflammatory disorders^12,13^. In encephalomyelitis (EAE), they mediate inflammation and demyelination by licensing myelin-specific T cell encephalitogenicity^14^.

In this study, we demonstrated that mast cells act in response to 3-IAld by up regulating Trp metabolism and releasing serotonin or 5-hydroxytryptamine (5-HT) (Tph1 pathway). Following this reasoning, we predicted that the microbial metabolite indole-derivative 3-IAld, induces a regulatory network through AhR in MS, where mast cells, by secreting the neurotransmitter 5-HT, contribute to maintain peripheral tolerance. Our findings thoroughly provide a molecular mechanism that explains how nuclear xenobiotic receptors regulate the immunomodulatory function of microbial metabolites via mast cells in autoimmunity.

## Results

### 3-IAld is detected in human serum and binds AhR

**Figure 1:**
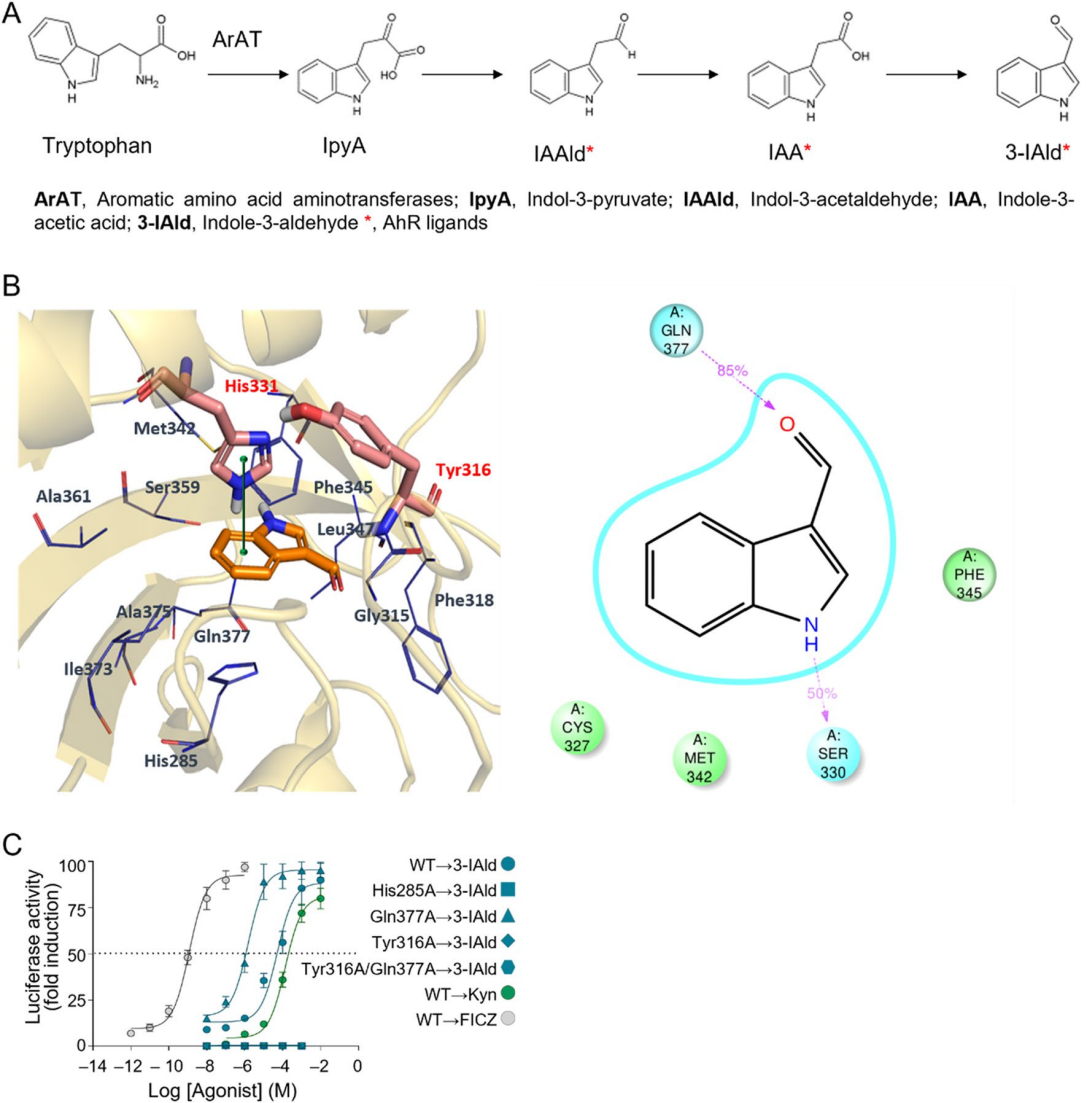
The amount of the blood circulating AhR ligand 3-IAld inversely correlates with MS duration. (**A**) The aromatic amino acid aminotransferase (ARAT) branch of the Trp metabolic pathway. Asterisks indicate metabolites with known AhR binding affinity. (**B**) Left panel: proposed binding mode of 3-IAld (orange carbon atom sticks) into the homology model of the PAS-B domain of the AhR; π–π stacking contacts between the indole ring and His331 are depicted with a continuous green line; right panel: schematic cartoon showing stable hydrogen bond interactions in magenta dashed lines with relative occupancy values as resulting from the MD simulation. (**C**) Transactivation activity of WT or Q377A, H285A, Y316A, Y316A/Q377A AhR by 3-IAld, in transfected MEFs. L-Kyn and FICZ were only challenged against the WT AhR.

Although the gut microbiome is deeply studied in MS^15,16^, the precise role played by systemic circulating microbial metabolites or postbiotics is poorly described. It has been shown that microbial propionic acid has a beneficial effect on immunological, neurodegenerative, and clinical parameters in MS patients^17^. We have focused on the levels of circulating microbial IPyA metabolites (Fig. 1A) in autoimmunity^18^. Among the indoles from the IPyA route, 3-IAAld was not detectable in human serum. Recent studies described the occurrence of this metabolite only in saliva and urine. Thus, we first investigated systemic 3-IAld and IAA levels in 35 patients with MS (Fig. S1A and Table S1). Serum 3-IAld and IAA levels did not differ according to sex, age, MS type, treatment status, MS disease activity or EDSS scores (Fig. S1A and data not shown). Indeed, although the bacterial communities in subjects with relapsing remitting MS have a lower abundance of *Lactobacilli* as compared to healthy controls^19^, upon using a previously published metagenome dataset that compared healthy subjects and MS patients^20^, we verified that 3-IAld producers (*Arat*^+^), were not less abundant in MS patients than in controls (Fig. S1B). However, it is worth to mention that the cohort presented by this study is almost entirely Caucasian with an increased proportion of males and a moderate number of patient recruited, which can be a limitation of this study. In addition, all MS patients had relapsing–remitting disease but none had an active relapse at the time of study enrollment. No other forms of MS were present in patients recruited^20^.

We focused our subsequent studies on 3-IAld, which also binds AhR more efficiently than IAA^3^, despite lower urine and serum concentrations than IAA in both humans (Fig. S1C) and mice (Fig. S1D). In addition, 3-IAld levels and not IAA did inversely correlate with MS disease duration in our cohort (Fig. S1E). This inverse correlation of 3-IAld with disease duration implies a possible role for gut dysbiosis and perturbation of postbiotic biological activity along the chronic biological course of human MS.

We first investigated whether 3-IAld could bind to the receptor interacting with the same host AhR fingerprint residues as the host-derived AhR ligand l-Kynurenine (l-Kyn)^21^. At this aim, we combined a docking study with molecular dynamic simulation, using an AhR PAS-B domain homology model (Fig. 1B)^21^. To substantiate our in silico findings, we observed that 3-IAld may indeed interact with AhR forming hydrogen bonds and π–π stacking contacts with polar and aromatic residues, respectively. We identified Gln377, Ser330, Tyr316 and His285 as key residues involved in these interactions (Fig. 1B and Fig. S2A). Notably, Tyr316 and Gln377 are necessary for L-Kyn–AhR binding also^22^. Trajectory (time: 100 ns) of 3-IAld (orange carbon atom sticks) into the homology model of the PAS-B domain of the AhR as resulting from the MD simulation, is shown in Supplemental Video1.

To substantiate the in silico data, we engineered a murine AhR wild type (WT) in which we mutated specific residues identified in AhR-3-IAld docking studies: Q377A, H285A, Y316A, Y316A/Q377A. We then reconstituted AhR-deficient mouse embryonic fibroblasts (MEFs) with murine WT or murine mutated AhR (Fig. 1C) or human wild type (WT) AhR (Fig. S2B-C). Then, we assayed AhR target gene *Cyp1a1* expression by adding 3-IAld, and found that MEFs reconstituted with H285A, Y316A or Y316A/Q377A AhR lost 3-IAld biologic activity in terms of potency and likely affinity, compared to WT AhR counterparts (Fig. 1C). Interestingly, opposite to L-Kyn, 3-IAld exhibited higher affinity for the Q377A AhR compared to WT, resulting increased reporter activity (Fig. 1C)^22^. While the 50% maximal effective concentration (EC50) value of 6-formylindolo[3,2-b]carbazole (FICZ), a strong AhR ligand, is lower than 3-IAld, the EC50 of 3-IAld is in the same range of L-Kyn^22^. Similarly, the AhR strong ligand 2,3,7,8-tetrachlorodibenzo-*p*-dioxin (TCDD) requires Y316 because of aromatic interactions, and H285A for electrostatic stabilization for the binding to mammalian AhRs^23^. Thus, Y316 or H285A AhR mutants completely perturbed the ability of TCDD to induce AhR nuclear translocation. The AhR mutation H285A, is known to also disrupt FICZ and L-Kyn binding to the the AhR ligand-binding pocket^24^. Among these known ligand-interactions to AhR, it is also interesting to mention 2-(1'H-indole-3'-carbonyl) thiazole-4-carboxylic acid methyl ester (ITE), known for nontoxic, immunomodulatory, and anticancer AhR-mediated functions. H285A is mostly involved in π–π stacking interactions with ITE thiazole or indole rings. Both H285A and Y316 AhR mutants do not show the ability of ITE to induce AhR translocation^25^. Those studies underline the fact that 3-IAld is binding the AhR binding site very similarly to known ligands.

### 3-IAld induces 5-HT release upon systemic administration

**Figure 2:**
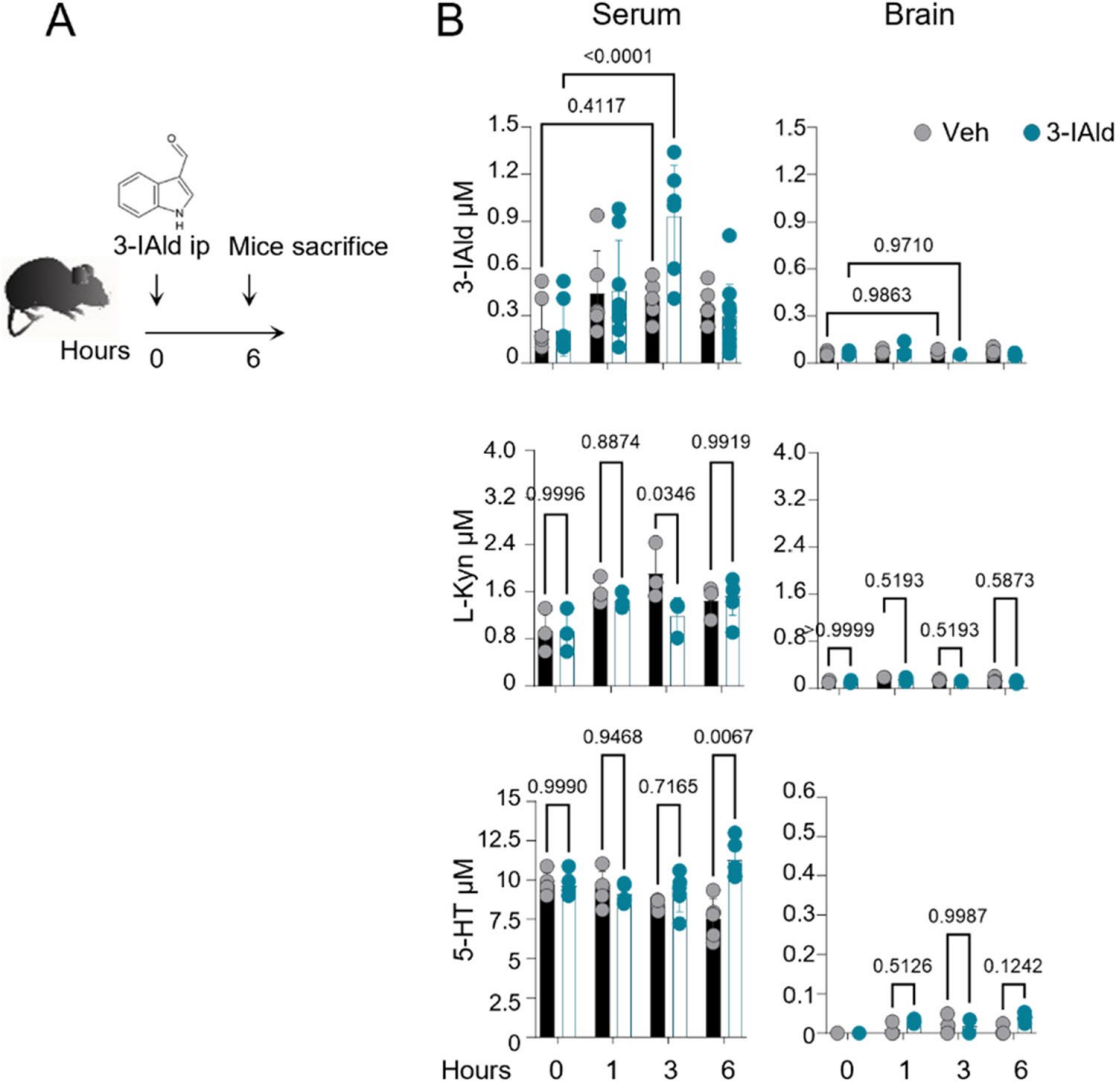
Systemic 3-IAld activates the Trp metabolism by enhancing the metabolic Tph1 peripheral pathway. (**A**) Mice were injected (i.p.) with 3-IAld or vehicle (Veh, DMSO 0.1% of olive oil) and Trp metabolites were quantified in serum and brain 1, 3 and 6 h after injection. (**B**) Trp metabolites and 3-IAld levels in murine serum and brain. Statistical analysis was performed using a Two-way ANOVA, Bonferroni post hoc test (**B**); (Vehicle, Veh (DMSO 0.1%)). ∗*p* < 0.05, ∗∗*p* < 0.01, ∗∗∗∗*p* < 0.0001. Data represent the means ± SD from three independent experiments.

Given the role of AhR activation as an important factor in controlling immunity, tissue homeostasis^26^ in inflammation^27^ and cancer^28^, we next treated mice systemically with 3-IAld and monitored its levels in various tissues by targeted metabolomics (Fig. 2A,B). We detected a physiologic peak of 3-IAld levels in the serum 3 h after treatment but found low levels in the brain not significantly different between treated and untreated groups (Fig. 2B), as well as in the lung or gastrointestinal tract (data not shown). Interestingly, we also found that, rather than promoting the IDO pathway, as demonstrated for L-Kyn^22^, 3-IAld would in fact tip the balance in favor of the serotonin (5-HT) metabolic branch (Fig. 2B). This metabolic switch is particularly evident in the serum of treated mice as shown in Fig. 2B. Most likely, we were not able to demonstrate this switch in the CNS, in part because of the very low levels of 3-IAld in the brain (Fig. 2B), but also because of the experimental difficulty of having a minimum timepoint inside the CNS 5-HT half-life.

We focused therefore on peripheral levels of 5-HT. 5-HT is synthesized along the tryptophan hydroxylase 1 (Tph1) pathway by specialized enterochromaffin cells (ECs) and mucosal mast cells^29^. Tph1 is also expressed on Th0, Th1, Th2 and iTreg-polarized cells^30^. More recently, it was also shown that Tph1 was induced in response to IL-2 in T cells in tumor microenvironment, leading to AhR nuclear translocation^31^.

### 3-IAld administration induces 5-HT release in mast cells via AhR and Tph1

**Figure 3:**
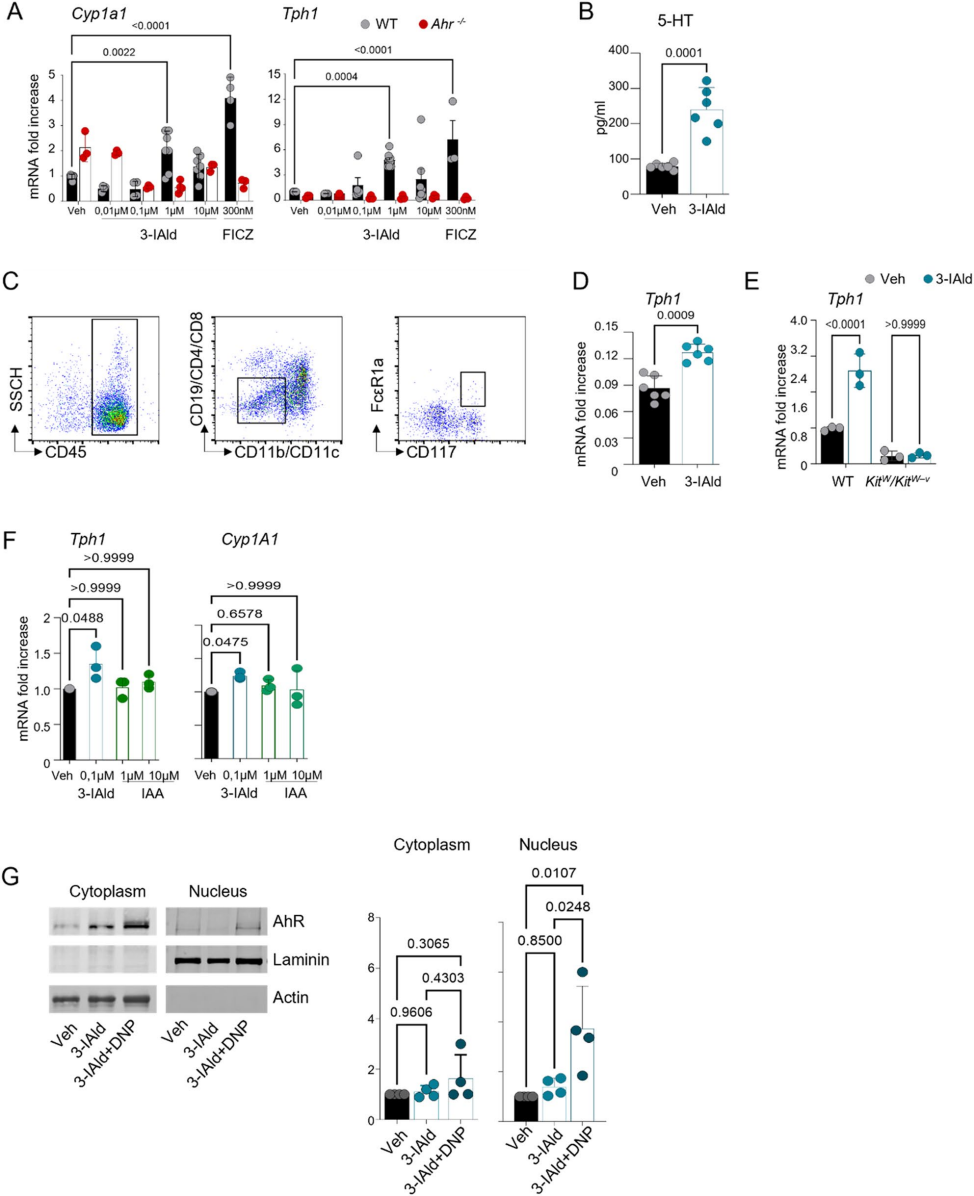
3-IAld activates the Trp metabolism by enhancing the metabolic Tph1 peripheral pathway in mast cells. (**A**) *Cyp1a1* and *Tph1* mRNA expression in WT and *Ahr*^−/−^ BMDMCs. (**B**) 5-HT release in WT BMDMCs. (**C**) WT ex vivo sorted mast cells were analyzed for *Tph1* mRNA expression (**D**) and *Tph1* mRNA expression (**E**) in WT and *Kit*^*W*^*/Kit*^*W–v*^ lymph node total cells. (**F**) *Cyp1A1* and *Tph1* mRNA expression in human CD34-derived mast cells. (**G**) Cytoplasmic and nuclear AhR translocation in WT BMDMCs exposed to 3-IAld or 3-IAld and DNP for 1 h. (Vehicle, Veh (DMSO 0.1%)). Statistical analysis was performed using Two-way ANOVA, Bonferroni post hoc test (**A**, **E**); Two-tailed Student’s t test, nonparametric Mann–Whitney U test (**B**, **D**); One-way ANOVA, Bonferroni post hoc test (B); ∗*p* < 0.05, ∗∗*p* < 0.01, ∗∗∗*p* < 0.001, ∗∗∗∗*p* < 0.0001.

We evaluated *Cyp1a1* and *Tph1* up-regulation in AhR-expressing mast cells, CD4^+^ T cells, and gut organoids containing ECs (*Lmx1a*^+^) as the main 5-HT producers, in response to 3-IAld (Fig. 3A and Fig. S3A-D). In bone marrow derived mast cells (BMDMCs), *Tph1* expression was induced via AhR (Fig. 3A) but only after IgE pre-incubation of mast cells; interestingly, *Ahr* deficient BMDMC when stimulated with 3-IAld (1 µM) downregulate significantly *Cyp1a1* expression (Fig. 3A). This result suggests that 3-IAld may bind other xenobiotic receptors (XRs) in the absence of AhR as for example PXR, affecting *Cyp* expression as already mentioned for the Indole 3-propionic acid^32^. In this regard, 3-IAld (from 1 to 100 µM) is not able to bind to murine PXR (data not shown), thus we can exclude on mast cells a role for PXR. Interestingly, the absence of AhR in mast cells alter STAT regulation as already demonstrated^33^, thus the high background seen in Fig. 3A may be explained by considering the AhR regulatory function also shown in macrophages^34^.

*Tph1* expression was induced upon 3-IAld treatment only after IgE exposure on BMDMCs (Fig. 3A and Fig. S3B). Interestingly, 1 µM 3-IAld increased 5-HT release from BMDMCs (Fig. 3B) and *Tph1* expression in sorted ex vivo mast cells (Fig. 3C,D, Fig.S3E). More importantly, *Tph1* expression was not induced in mast-cell-deficient *Kit*^*W/*^*Kit*^*W–v*^ mice compared to WT littermates (Fig. 3E) at 6 h post administration, in peripheral lymph nodes.

We then evaluated *Cyp1a1* and *Tph1* up-regulation in human IgE-treated CD34-derived mast cells upon exposure to AhR ligands (Fig. 3F). Comparing the ligand doses able to activate the AhR-Cyp1A1 axis in murine mast cells (Fig. 3A), with the concentrations able to activate this axis in human mast cells (Fig. 3F) revealed that human cells are more sensitive to AhR ligand lower concentrations. At 0.1 µM, 3-IAld induces *Tph1* and *Cyp1A1* (Fig. 3F). Human mast cells were not sensitive to IAA, although it is more abundant in human serum (Figs. 3F and S1C). *Cyp1a1*, not *Tph1* was induced in CD4^+^ T cells (Fig. S3A).

In conclusions, results here show that the induction of both *Cyp1A1* and *Tph1* in human and murine mast cells occurs after IgE binding to Fc ∊ RI and exposure to 3-IAld. To confirm that *Cyp1A1* is effectively due to AhR activity in mast cells, we have demonstrated nuclear AhR translocation by western blotting in mast cells while exposed to 3-IAld (Fig. 3G).

### 3-IAld activates a mast cell tolerogenic program via AhR and Tph1

**Figure 4:**
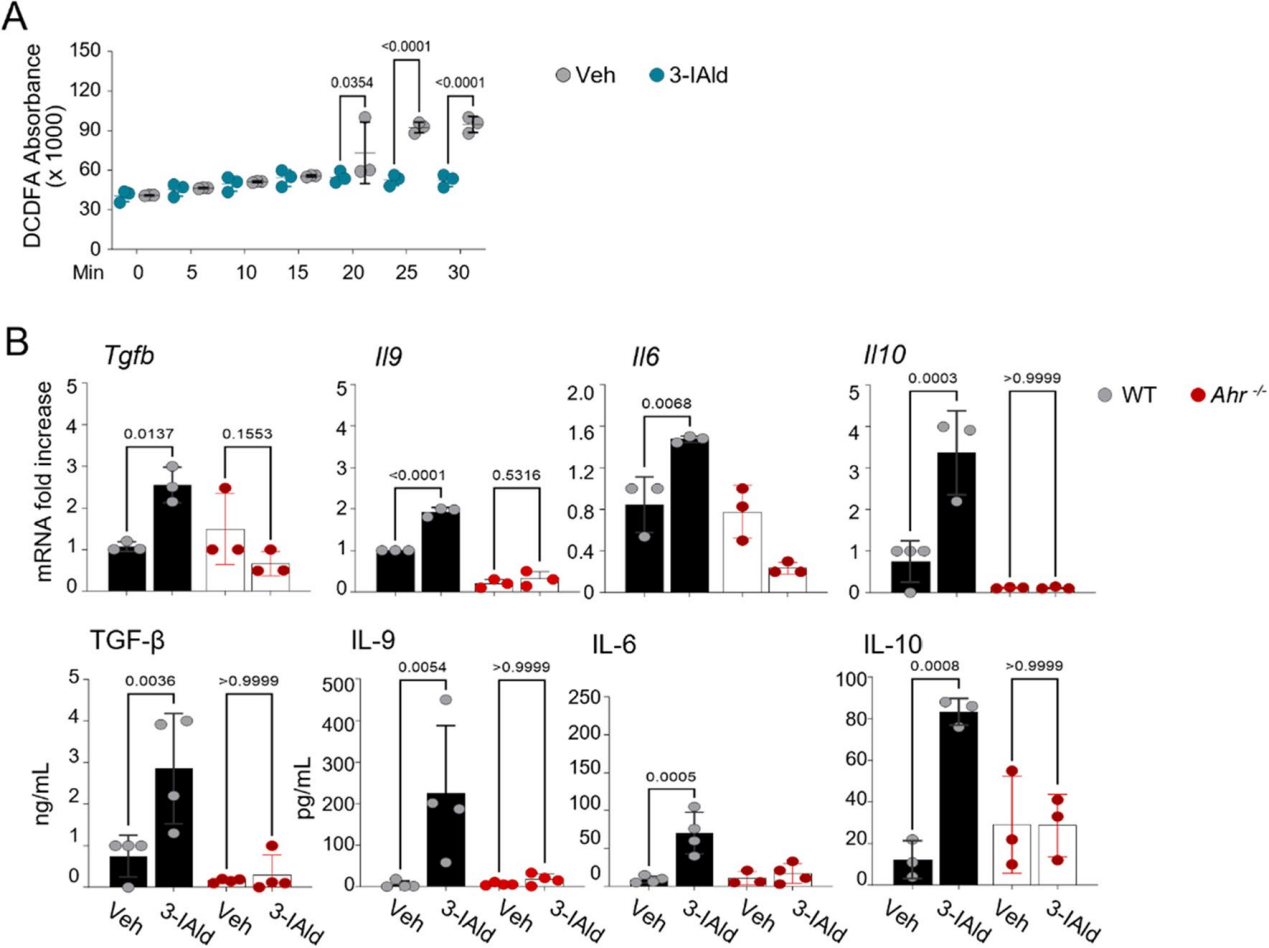
3-IAld activates a mast cell tolerogenic program via AhR and Tph1. (**A**) Reactive Oxygen Species (ROS) detected in WT BMDMCs. (**B**) Real-time PCR and ELISA of the indicated targets in WT and *Ahr*^−/−^ BMDMCs (Vehicle, Veh (DMSO 0.1%)). Statistical analysis was performed using Multiple T tests (**A**), Two-way ANOVA (**B**), Bonferroni post hoc test. *n.s.*, not significant. ∗*p* < 0.05, ∗∗*p* < 0.01, ∗ ∗ ∗*p* < 0.001, ∗∗∗∗*p* < 0.0001.

To deeply investigate on the effect of 3-IAld on mast cells, we have measured BMDMC activation, and we found that 3-IAld did not modulate cell degranulation (Fig. S4), but rather decreased ROS release (Fig. 4A) and modulated cytokine production (Fig. 4B) in AhR-dependent manner. Remarkably, and like L-Kyn, 3-IAld induced *Tgfβ* and *Il10* mRNA expression and protein release*.* As previously reported for other AhR ligands^35^, 3-IAld-induced BMDMC activation increasing IL-9 production*,* a key mediator of mast cell recruitment^36^, but differently from monocytes^37^, IL-6 expression and release in AhR-dependent manner (Fig. 4B).

Although controversial, it has been already reported that *Ahr* deficient BMDMC do not show alterations of baseline activities as shown for degranulation upon antigen recognition or Ionomycin treatment (Fig. S4)^33,35^.

Because mast cells and T cells are often found in proximity in secondary lymphoid organs and specific sites of tissue inflammation, it is not surprising that their interactions affect the functional capabilities of both cell types^38–40^. Indeed, we observed that after 3-IAld treatment, mast cells expressed all the relevant cytokines (IL-6, IL-10 and TGFβ) that are known to regulate adaptive immunity.

### Systemically administered 3-IAld protects from EAE by activating AhR and Tph1 in CLN mast cells

**Figure 5:**
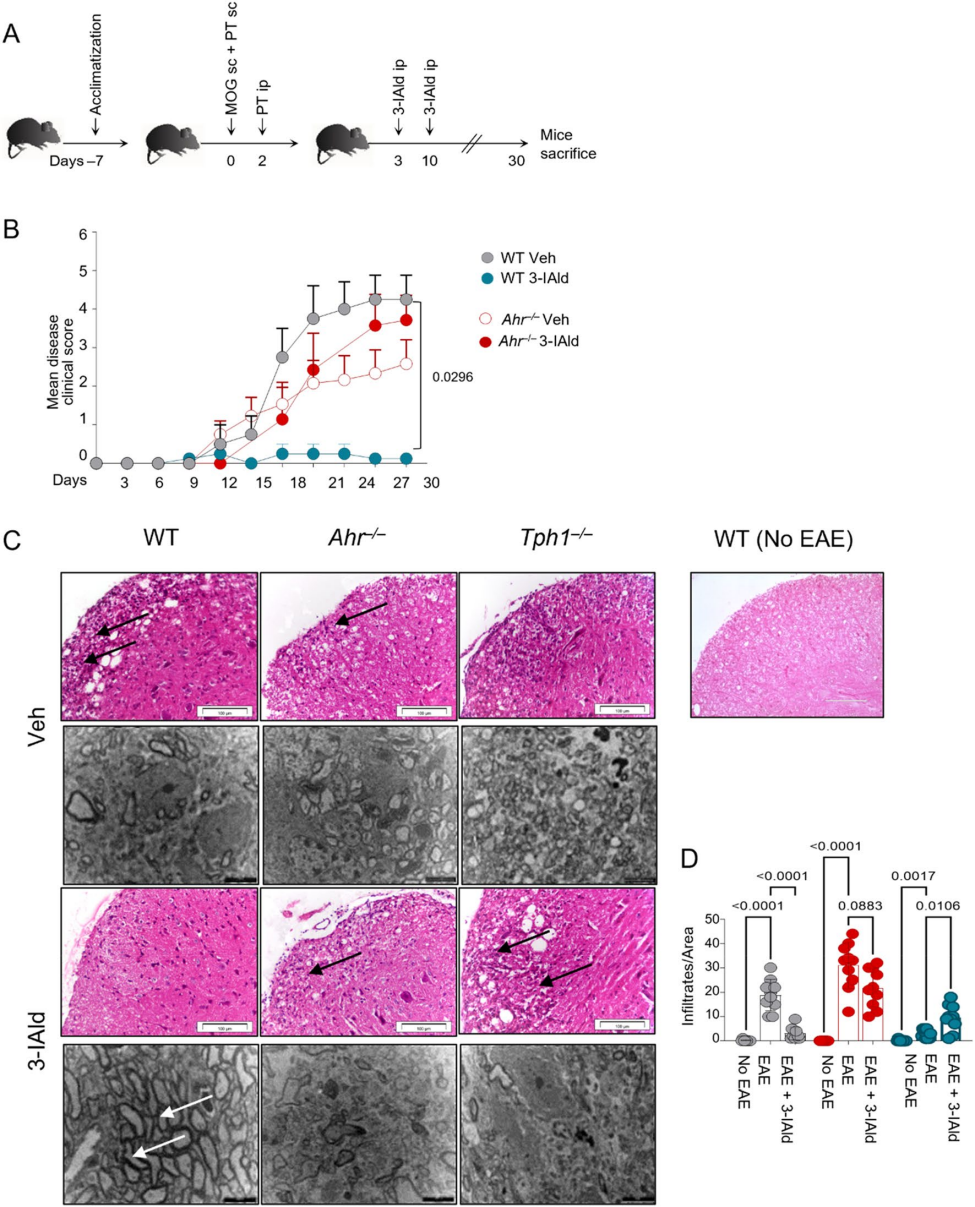
3-IAld systemic treatment protects mice from EAE inducing the AhR-Tph1-mast cell axis. (**A**) EAE was induced in WT, *Ahr*^−/−^*, Tph1*^−/−^ mice, 3-IAld (0.36 mg/mouse in olive oil) or Veh (DMSO 0.1% of olive oil) were injected i.p. at the indicated time points. (**B**) Mean clinical score registered every 2 days in WT, *Ahr*^−/−^*, Tph1*^−/−^ treated and untreated mice. (**C**) Cerebellum slice, viewed by transmission electron microscopy (scale bar = 5 µm) and hematoxylin and eosin stain on paraffin sections of spinal cords. Arrows indicate inflammatory infiltrates (scale bars = 100 μm). Assays were performed at 30 dpi and data were pooled from four independent experiments (*n* = 8 mice per group); mean ± SEM. (**D**) Quantification of inflammatory infiltrates in WT, *Ahr*^−/−^*, Tph1*^−/−^ murine spinal cords with EAE and without EAE. Right panel, spinal cord hematoxylin and eosin paraffin section histology of non-EAE mice (scale bar = 50 μm). Statistical analysis was performed using a Multiple T test (**B**); Two-way ANOVA, Bonferroni post hoc test (**D**). ∗∗*p* < 0.01, ∗∗∗*p* < 0.001, ∗∗∗∗*p* < 0.0001.

5-HT can attenuate cytokine production by effector T cells in vitro and favor anti-inflammatory responses in MS patients^41^. In view of that, we established EAE in WT, *Ahr*^−/−^, *Tph1*^−/−^ and treated the animals with 3-IAld or vehicle control (Fig. 5A, S5). In response to 3-IAld, we observed minimal EAE progression in WT mice that eventually disappeared (Fig. 5B–D) and no evidence of myelin loss or inflammatory infiltration in the spinal cord (Fig. 5C). By contrast, we observed an evident pathologic condition of untreated WT and treated and untreated *Ahr*^−/−^ mice (Fig. 5B), characterized by high inflammatory infiltration and demyelination plaques in the spinal cord (Fig. 5C,D) and myelin loss in the cerebellum evaluated by TEM (Fig. 5C, lower panels). We confirmed that 3-IAld mast cells play a central role in the effect observed upon 3-IAld treatment, using a reconstitution assay of *Kit*^*W/*^*Kit*^*W–v*^ mice, which per se exhibited delayed EAE onset than their mast cell-competent WT littermates as shown already^42^. Eight weeks after reconstituting *Kit*^*W/*^*Kit*^*W–v*^ with mast cells, 3-IAld administration reduced EAE clinical signs in comparison with non-reconstituted *Kit*^*W/*^*Kit*^*W–v*^ mice (Fig. S5A,B). Because of the low solubility of 3-IAld in water, we administered the molecule in DMSO 0.1% in olive oil (vehicle), as it was shown for other AhR ligands administered systemically^43^. Thus, we have compared mice treated with 3-IAld with mice administered with vehicle only to better appreciate the effects due to 3-IAld administration., since it is well known how plant oils may impact differentially on EAE induction^44^.

We also observed concomitant AhR and 5-HT pathway activation in cervical lymph nodes (CLN), indicated by *Cyp1a1* and *Tph1* increased expression upon 3-IAld treatment (Fig. S6A). In terms of T cell cytokine signature induced, 3-IAld was able to increase in CLN *Il10* and *Il17* mRNA (Fig. S6B). Finally, 3-IAld treatment in *Tph1*^−/−^ mice did not reduce EAE clinical scores (Fig. S5C) but strongly promoted *Il17* expression in CLN (Fig. S6B). This result may explain the reason why 3-IAld exacerbates EAE symptoms in treated *Tph1*^−/−^ mice (Fig. 5C,D and Fig. S6C).

Interestingly, the induction of *Tph1* and *Cyp1a1* expression was not occurring in the spinal cord (Fig. S6C) upon 3-IAld treatment as well as the 5-HT release (data not shown). Also, *Il17a* and *Il-10* mRNA levels were not induced in 3-IAld mice group in spinal cord (Fig. S6D), although 3-IAld modulated cytokine release in spinal cord and in cervical lymph nodes (Fig. S6E,F).

**Figure 6:**
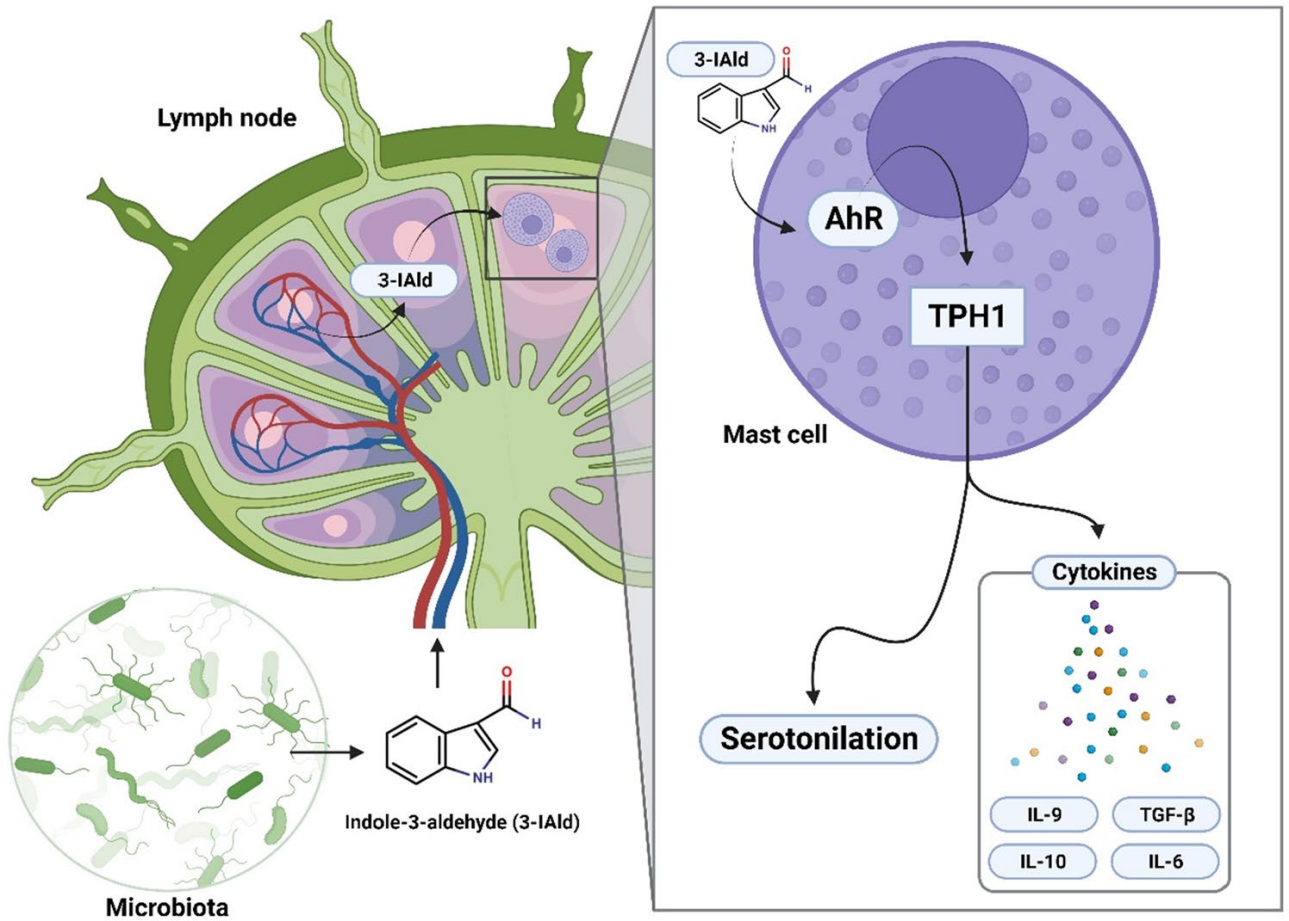
3-IAld affects the cytokine microenvironment in peripheral lymph nodes via Tph1. 3-IAld is a tryptophan-derived indole metabolically released by several components of the gut microbiome. We have shown how the systemic administration of the indole-derivative activates the enzyme Tph1 in mast cells, in the lymph node compartments. The activation of the Tph1-mast cell axis is mediated by the binding of the nuclear receptor AhR. This mechanism supports how the administration of 3-IAld affects systemic inflammation in a mouse model of EAE and suggests that changes in serum concentration of indole-derivatives may affect the overall immunity.

These results together show that 3-IAld systemically administered, activates in peripheral lymph nodes a mast cell signaling pathway dominated by Tph1, which may in turn affect peripheral tolerance through serotonilation. Collectively, these findings support the hypothesis that mast cells expressing Tph1 signalling, play a role in the peripheral tolerance of MS as already demonstrated following NAD^+^ exposure to mast cells^45^. Thus, we propose a novel model of action of postbiotics binding AhR, circulating in blood and lymph nodes (Fig. 6).

## Discussion

In previous studies, 3-IAld has been administered in drinking water or as a food supplement to protect from DSS-colitis^3^ and EAE, respectively^8,46^; in vitro*,* 3-IAld has been used to reprogram gut intraepithelial CD4^+^ T cells into immuno-regulatory T cells^47^. Here we administered 3-IAld systemically, to understand the impact of this immunomodulatory postbiotic on systemic inflammation. We conclude that 3-IAld acts as a typical AhR ligand and induces a regulatory network in MS following AhR binding, whereby mast cells secrete 5-HT to help maintain self-tolerance-expanding regulatory T cells.

This effect is highly reasonable, as indeed dietary and microbial metabolites, particularly Trp metabolites, may have an important anti-inflammatory role in the CNS^48^. The AhR plays a key role in affecting immune responses in autoimmune diseases. On microglial cells, in EAE mouse model, activation of AhR results in a limitation of pro-inflammatory transcriptional responses, reducing pathogenic inflammatory activities^49^. On T cells, in vitro, it has been shown to increase IL-17/IL-22 production. On the other side, the use of conditional AhR-deficient mice, showed that the anti-inflammatory effect of AhR ligands strongly depends on AhR activation in vivo in both T cells and dendritic cells^43^. More recently, conclusively, AhR is associated to non-pathogenic T cells in EAE^50^. This complexity may explain the reason why we don’t see a significant susceptible or resistant phenotype of AhR deficient mice to EAE compared WT animals.

Specifically, we found that systemic 3-IAld led to mast cell plasticity towards an anti-inflammatory phenotype in peripheral lymph nodes that may suppress Th17 pathogenicity in autoimmunity^51,52^.

Remarkably, this mechanism not only necessitates AhR activation, but is mediated by the 5-HT pathway that is activated in mast cells. Similarly, it has been shown in CD8^+^ T cells that AhR translocation in the tumor microenvironment, induces 5-HT release leading to CD8^+^ T cells exhaustion. To further substantiate our results, it has been shown that 5-HT reduced IL-17 release in Th17 in MS patients^53^. Consistently, 3-IAld serum levels are inversely correlated with disease duration in MS, implicating that chronic inflammation may affect postbiotic physiological systemic concentration, thus exacerbating the pathogenic scenario in autoimmunity.

In conclusion, we have here demonstrated that because of the spatial separation between CNS and periphery, attributable in this context to the inability of 5-HT to cross the blood–brain barrier, the postbiotic 3-IAld acts only peripherally on AhR mast cells upon IgE sensitization. Mast cell-Tph1 is not affecting the neuronal 5-HT amount, known to modulate sleep, mood and appetite, but is affecting the peripheral 5-HT known to regulate gastrointestinal motility, liver regeneration, bone formation, and immune responses through a mechanism also known as ‘serotonilation’. The concept that mast cells activate several biological outcomes only upon IgE sensitization is not novel and it was indeed extensively demonstrated in the last years in different pathologies^54^. Furthermore, there is the evidence of mast cell activation in MS, which dates back to more than a century^55^. Therefore, it is likely that in MS, mast cells are sensitized by circulating IgE affecting the disease pathogenesis on the basis of the degranulation rate.

In EAE, we show that by enriching the lymph node microenvironment of 5-HT, inflammation is affected. This is also plausible since T cells may respond to 5-HT via 5-HT receptors. Treg cells express 5-HT2R as recently demonstrated^56,57^. Thus, we propose that postbiotics may act on 5-HT peripheral levels, differently from classical drugs as monoamine oxidase inhibitors. Mast cell activation and migration to lymph nodes during induction of an immune response in mice has been already shown in a model of mice hypersensitivity^58^. These results may eventually help to understand how in MS, T cells are affected in their differentiation in periphery while inflammation is occurring in the spinal cord.

Finally, the development of postbiotics-targeted therapies is an intriguing new opportunity for many diseases. Several studies recently show how different therapy based on microorganisms may affect MS disease outcome^59^. However, this approach led to controversial results as shown in particular for the use of lactobacilli^59–63^.

Our experimental data in mice and human suggest a novel approach based on microbiome products more than on microbiome individual composition. The identification of 3-IAld as a ‘natural AhR ligand’ could serve as a basis for the development of novel prevention and treatment strategies in MS.

## Methods

### Mice

C57BL/6 and breeder pairs of *Kit*^*W*^*/Kit*^*W−v*^ mice were purchased from Charles River Laboratory. *Tph1* knockout mice (*Tph1*^−/−^) were supplied by Prof Dr. Michael Bader (Max-Delbruck Centrum for molecular medicine. Berlin, Germany). 129-Ahrtm1Bra/J mice deficient for *Ahr* (*Ahr*^−/−^), were supplied by Prof. Francesca Fallarino (University of Perugia, Dep. of Experimental Medicine. Perugia, Italy). Mice were housed in a controlled environment at the University of Perugia animal facility and were provided with standard rodent chow and water.

All experimental protocols were approved by the Ministry of Health (Authorization N. 862/2017-PR, on November 3rd 2017) and previously certified by the ethic animal committee ‘OPBA’ from the University of Perugia. All methods were conducted in accordance with relevant guidelines and regulations. All methods are reported in accordance with ARRIVE guidelines (https://arriveguidelines.org).

### Docking study and molecular dynamic simulation

A multiple alignment between the PAS-B mAhR (residues 278–384) and PAS-B HIF-2α sequences was generated according to the sequence alignment suggested by Pandini et al.^23^ The homology model of PAS-B mAhR was constructed using the crystal structures of the PAS-B HIF-2α heterodimer complex (pdb code: 3f1p, 3f1o, 3f1n) and the multiple-template approach implemented in Modeller^64^. 3-IAld was built using the ligand preparation tool (LigPrep) of Maestro. Docking experiments were carried using Glide and the QM-polarized ligand docking procedure (QPLD), following our previously reported computational protocol^25^. The best energetic scored solution was further refined using molecular dynamic (MD) simulation. Briefly, an orthorhombic box of TIP3P water molecules was used to solvate 3-IAld/AhR receptor complex, with an edge extension of 10 Å away from any protein atom. The resulting system was neutralized by adding sodium and chlorine ions at a concentration of 0.15 M. Periodic boundary conditions were then applied to avoid finite-size effects. Atomic partial charges of 3-IAld generated in QPLD calculation were kept for the MD simulation. Desmond (Schrödinger Release 2014-4) and the OPLS-2005 force field were employed for the MD simulation. The simulation protocol included starting relaxation steps and a final production phase of 100 ns. The occupancy of intermolecular hydrogen bonds, aromatic interactions and hydrophobic contacts was calculated along the trajectory produced by the MD simulation, using the Simulation Interaction Diagram Tools implemented in Maestro. A cut-off value of 30% of interactions fraction was used to select the most relevant interactions for 3-IAld and key residues in AhR binding site (Fig. 1C and Fig. S2A).

### Luciferase Assay

Mouse embryonic fibrobasts (MEF) (2 × 10^5^) were electroporated (230 V, 75 Ohm and 1,500 microfarads) with 2 μg WT or AhR mutant plasmid (H285A), (Q377A), (Y316A), (Y316A/Q377A) in Optimem/Glutamax (Invitrogen) with 0.8 μg firefly luciferase reporter pGudLuc1.1 plasmid, which contains a 480 bp fragment of the upstream enhancer region of the mouse *Cyp1a1* gene—including four xenobiotic response elements—upstream of the firefly luciferase coding sequence. The pRL-TK (0,2 μg; Promega) reporter plasmid encoding Renilla luciferase was electroporated as an internal control of the transfection process. Cells were seeded in 24-well plates at a density of 2 × 10^5^ cells/ml. After 24 h at 37 °C, cells were stimulated for 6–8 h with increasing concentrations of 3-IAld. Since 3-IAld was dissolved in DMSO (present as 0.1% *v/v* of experimental medium), 0.1% of DMSO was chosen as a vehicle. Luciferase assays were performed using the dual luciferase reporter assay kit (Promega).

### Sample collection and preservation

All study contributors were required to fast overnight. Urine and fasting blood samples (5–7 ml) were collected from MS patients and healthy subjects into separating tubes. Peripheral venous blood (3 − 5 ml) was obtained from healthy donors and MS patients. Human blood samples were kept at room temperature for 20 min, allowing the coagulated blood to clot, and then centrifuged at 750 × g for 15 min to yield the serum. The urine samples were separated by centrifuging at 1200 × g for 20 min and were stored at − 80 °C until biochemical analysis.

### 3-IAld biodistribution study

A total of 18 C57BL6 mice were used (*n* = 3/time point/group): the groups represented the vehicle (Veh) control (DMSO 0.1% of olive oil) and 3-IAld (Sigma Aldrich) treatment. A time course study (1, 3, 6 h) was carried out to characterize the biodistribution of 3-IAld and Trp metabolites in brain and serum after ip. injection. Veh or 3,6 mg/mL of 3-IAld were injected i.p. in a single volume of 100 μl. At the appropriate time points, animals were sacrificed for organ dissection and blood was drawn.

### HPLC–MS/MS analyses

For human HPLC–MS/MS analyses, urine and serum were obtained from healthy subjects and MS patients. For murine analyses, urine, serum and brain homogenate samples were harvested from mice housed in a standard metabolic cage.

#### Chemicals and reagents

All solvents and reagents were LC–MS grade and were supplied from Sigma-Aldrich (Milan, Italy). A 1 mg/mL stock solution of the isotopically-labeled internal standard D7-indole-3-acetic acid (isotopic enrichment 97–98%, CIL) was prepared in methanol (MeOH) and stored at − 80 °C. Individual 1 mg/mL stock solutions of unlabelled analytes, indole-3-carboxaldehyde (purity 97%, Sigma-Aldrich) and indole-3-acetic acid (purity 99%, Sigma Aldrich) were also prepared in MeOH and stored at − 80 °C. Three separate working standard solutions were prepared, one containing the isotope-labeled standard at 60 µg/mL in MeOH, one containing the, indole-3-acetic acid at 60 µg/mL in MeOH and one containing indole-3-carboxaldehyde at 6.0 µg/mL, and stored at − 80 °C. The standard solutions and the isotope-labeled standard solution were freshly diluted 24 times in water with 5 mM ammonium acetate (AmAc) and 0.2% formic acid (FoAc) (eluent A) and used to construct the calibration curve (7 points) in the range of concentrations of interest for quantitative measurements. For human and mouse sera, the calibration curve covered the range from 0.38 to 20.00 µM, while in urine was from 0.82 to 47.5 µM, corresponding to 20 pg/µL and 1050 pg/µL, respectively, in the solution injected in the LC–MS/MS for indole-3-acetic acid. A linear calibration curve was obtained (y = 0.918 x + 0.072, r^2^ 0.991), using a 1/x weighting factor. For indole-3-carboxaldehyde, the calibration curve covered the range from 0.02 to 2.20 µM, while in urine was from 0.05 to 5.2 µM, corresponding to 0.87 pg/µL and 96 pg/µL, respectively, in the solution injected in the LC–MS/MS. A linear calibration curve was obtained (y = 0.573 x + 0.009, r^2^ 0.989), using a 1/x weighting factor. A negligible matrix effect was calculated using the internal standard peak area, comparing the signal intensities obtained from spiking of D7-indole-3-acetic acid in 200 µL eluent A or in 200 uL of a sample treated as described later but adding the internal standard immediately before the injection, to obtain a final concentration of 75 pg/µL. D7-indole-3-acetic acid peak area in the sample matrix was in the range 93–96% of that in the HPLC eluent A, indicating a minimal ion suppression effect for human and mouse sera and urines. A higher suppression effect was recorded in brain homogenate surnatant, with a 9–13% reduction of the peak area value. For brain homogenate a calibration curve was prepared with indole-3-carboxaldehyde and indole-3-acetic acid both at concentration between 0.04 µM and 1.2 µM.

#### Samples preparation

Human serum (200 µL), mouse serum (200 µL) and mouse brain homogenate surnatant (300 µL) were spiked with 20 µL of the solution of internal standard (2.5 µg/mL) and diluted to 1.0 mL final volume with cold acidified methanol (MeOH; Sigma-Aldrich) in 1.5 mL Eppendorf vial. After centrifugation at 8000×*g* for 10 min, the solvent (300 µL) was transferred in a new vial for LC injector, taken to dryness using a vacuum evaporator. The residue was re-dissolved in 200 µL of HPLC eluent A and the sample analysed by HPLC–MS/MS.

Human urine and mouse urine (20 µl), to which 6 µL of the solution of internal standard (2.5 µg/mL) were added, were diluted to a final volume of 200 μL with HPLC eluent A and then injected in the HPLC–MS/MS instrument.

#### Liquid chromatography-tandem mass spectrometry

A Dionex Ultimate 3000 HPLC system was used (Thermo Scientific) coupled to an API 4000 Qtrap LC–MS/MS (AB Sciex, Toronto, Canada) equipped with a TurboV Ion Spray source operating in positive ion mode. Analyst software (v.1.6.2) from AB Sciex was used for data acquisition and analysis. All MS parameters were optimized by direct infusion of solution of each analyte and source parameters (gas flows and temperatures) by flow injection. The ion source operated with spray voltage set at 5.3 kV, curtain gas at 24, ion source temperature at 550 °C, GS 1 70, GS 2 48; collision gas low. Analytes were detected using scheduled multiple reaction monitoring (MRM) acquisition, using 150 s detection window and 3 s target scan time; two transitions were monitored for each molecule. All the acquisition parameters are listed in the Tables S2 And S3. A Ultra AQ C18 column (100 × 2.1 mm, 3 µm; Restek, USA) was used; eluents were 5 mM AmAc in water (A) and acetonitrile (B), both containing 0.2% FoAc. The column temperature was kept at 35 °C. Chromatographic separation of the analytes was performed using a linear gradient; 10 µL injection volume was used. The column effluent was delivered to the mass spectrometer with no split.

### Generation of bone marrow derived mast cells (BMDMCs)

Mast cells were derived from bone marrow precursors by culturing stem cells flushed from tibia and femurs of 4-week-old female C57BL/6, *Ahr*^*−/−*^ and *Tph1*^*−/−*^ mice in RPMI 1640 supplemented with 20% fetal bovine serum (Euroclone), 2 mM glutamine (Sigma Aldrich), 100 U/ml penicillin streptomycin (Lonza), non essential amino acids (Sigma Aldrich), 1 mM sodium pyruvate (Sigma Aldrich), 20 mM HEPES (Sigma Aldrich), 5 ng/ml recombinant murine IL-3 (Peprotech) and 5 ng/ml murine stem cell factor (Milteniy) for 4 to 8 weeks. Cell cultures were grown at 37 °C in a humidified atmosphere with 5% CO_2_. Purity was measured by FACS by monitoring FcεRI and cKit receptor expression (> 90%).

### BMDMCs culture

BMDMCs were treated with IgE (monoclonal anti-DNP antibody, Sigma Aldrich; 1 μg/ml) overnight and treated with an increasing dosage of 3-IAld, 6-Formylindolo[3,2-b]carbazole (FICZ, Sigma Aldrich; 300 nM) or with (0.1% DMSO) as vehicle control. The following day, the cells were challenged with antigen-specific IgE (BSA-DNP, Thermo Scientific; 1 μg/ml) and cultured for 3 h. Following culture, the supernatants were collected and the cells were lysed with 1 ml TRIzol® reagent (Ambion, Life Technologies) and flashly frozen on dry ice prior to storage at − 80 °C.

### Naïve CD4^+^ T cell isolation and culture

Spleens from C57BL/6 WT mice were harvested and digested with pre-warmed HBSS containing Collagenase IV (Sigma-Aldrich; 400U/ml) and DNase I (Sigma-Aldrich; 30 μg/ml) and incubated at 37 °C for 40 min for enzymatic digestion. After blocking enzymatic activity with 10% FBS medium, splenocytes were isolated by magnetic cell sorting with CD4 (L3T4) MicroBeads (Miltenyi Biotec) following the manufacturer’s protocol. CD4^+^ T cells were triggered with 3-IAld (1uM) and cultured for 5 days at 37 °C, 5%CO_2_ in a 24-well plate with plate-bound αCD3 (10 μg/mL), αCD28 (20 μg/mL).

### RNA extraction and quantitative polymerase chain reaction (qPCR)

Total RNA was extracted from cells by using TRIzol® reagent (Ambion, Life Technologies). The cDNA synthesis kit (BioRad) was used for cDNA analysis. Real-time PCR was performed using SYBR Green qPCR master mix (BioRad). All reactions were performed at least three times independently. The PCR primer sequences (5’-3’) were as follows:

*β-actin:* AGCCATGTACGTAGCCATCC and CTCTCAGCTGTGGTGGTGAA.

*Il-10:* GAGAAGCATGGCCCAGAAATCAAG and ATCACTCTTCACCTGCTCCACTGC.

*Il-17:* GACTACCTCAACCGTTCCAC and CCTCCGCATTGACACAGC.

*Rorc:* ACAACAGCAGCAAGTGATGG and CCTGGATTTATCCCTGCTGA.

*Tph1:* GTCCCGGAAATCAAAGCAAAGA and GGGCGAGTCCACCGAGAGG.

*Cyp1a1:* ACAGTGATTGGCAGAGATCG and GAAGGGGACGAAGGATGAAT.

*Tgfb:* ATATTTGGAGCCTGGACACA and CGTAGTAGACGATGGGCAGT.

*Il-9:* TGACCAGCTGCTTGTGTCTC and GTGGCATTGGTCAGCTGTAA.

*Ahr:* TCCATCCTGGAAATTCGAACC and TCTTCATCCGTCAGTGGTCTC.

*Il-6:* CCGGAGAGGAGACTTCACAG and TCCACGATTTCCCAGAGAAC.

### Mast cell purification and culture

Ex vivo mast cells were enriched from popliteal and mesenteric lymph-nodes by negative selection with Gr1/Ly6G, CD3, CD45/B220 and Ter-119 microbeads (Milteny Biotec). Enriched mast cells were further purified with a cell sorter (MoFlo Astrios, Beckman Coulter) by gating on CD23 and CD117. Cell purity was > 95%. Mast cells were then cultured overnight and treated with 3-IAld or DMSO 0.1% as a vehicle control.

### ELISA

Murine cytokine TGF-β, GM-CSF, IFN-γ, TNF-α, IL-1-β (eBioscience), IL-9, IL-6 (Thermo Fischer Scientific) and 5-HT levels (TECAN) were determined by ELISA on the supernatants of cultured cells. Absorbance was measured using a TECAN microplate reader (Infinite M200).

### Intracellular reactive oxygen species (ROS) determination

IgE pre-sensitized BMDMCs were incubated with DNP- BSA (ThermoFisher Scientific; 1 µg/ml) for 30 min in the presence or absence of 3-IAld (1 μM) and DMSO 0.1% as vehicle control. Intracellular ROS were detected by incubating mast cells with the oxidation-sensitive fluorescent probe dichlorodihydrofluorescein diacetate acetyl ester (DCFH-DA, Sigma Aldrich; 5 µM) for 30 min at 37 °C. Cellular fluorescence intensity was measured using a TECAN microplate fluorescence reader (Infinite M200).

### CD34-derived human mast Cells

Primary human mast cells were differentiated as described in^65^. CD34^+^ hematopoietic progenitors were isolated from mobilised peripheral blood (Department of Transfusion & Tissue Medicine of the Brno University Hospital) using the CD34 MicroBead Kit UltraPure (Miltenyi Biotec) following manufacturer recommendation.

5 × 10^6^ CD34^+^ cells were seeded at a concentration of 10^5^ cells/ml in human mast cell media (StemPRO-34 (Gibco), 100 U/ml Penicillin, 100 ug/ml Streptomycin, 1 × GlutaMAX (Gibco), 100 ng/ml rhSCF (Peprotech), 100 ng/ml rhIL6 (Stemcell Technologies)). During the first week of culture, 100 ng/ml rhIL3 (Stemcell Technologies) were added to the media. Media was changed weekly by hemi-depletion and cells were maintained at a concentration below 5 × 10^5^/ml.

Cells were used for experiments after 8 weeks of culture.

### Cell stimulation

5 × 10^5^ primary human mast cells were seeded overnight in the presence of 1 µg/ml anti-DNP IgE (Sigma Aldrich). The next day, half of the media was carefully removed and cells were stimulated for 5 h with the ligands indicated in each figure at a final concentration of 10^6^ cells/ml before RNA isolation. Cells were then harvested, centrifuged at 350 g for 5 min and RNA was isolated by column separation using the RNeasy Mini Kit (Quiagen) following manufacturer recommendations.

### Gene expression assay

RNA was converted to cDNA with the High-Capacity cDNA Reverse Transcription Kit (Applied Biosystems) and used for qPCR. All qPCR assays were performed using TaqMan probes (CYP1A1—Hs00153120_m1, TPH1—Hs00188220_m1) and TaqMan™ Gene Expression Master Mix with a StepOne™ Plus Real-Time PCR System (Thermo Fisher Scientific).

### EAE induction and treatment

Mice aged 6–8 weeks were immunized subcutaneously (s.c.) at the base of the tail and under the neck with 300 μg pMOG_35–55_ (Cambridge Research Biochemicals) solubilized in Complete Freund’s Adjuvant (CFA; Sigma Aldrich). Mice also received 200 ng Bordetella pertussis toxin (Sigma-Aldrich) intraperitoneally (i.p.) on days 0 and 3. 3-IAld (Sigma Aldrich; 0.36 mg/mouse in olive oil) or DMSO 0.1% of olive oil (Sigma Aldrich) as vehicle control, were administered i.p. on days 3 and 11 post immunization (pi). EAE clinical assessment was performed every 2 days and clinical scores were assessed according to the following criteria: 0 = unaffected, 1 = flaccid tail, 2 = impaired righting reflex and/or gait, 3 = partial hind limb paralysis, 4 = total hind limb paralysis, 5 = total hind limb paralysis with partial fore limb paralysis. Mice were sacrificed on day 30 pi. Sections (4% PFA fixed) were stained with Hematoxylin and Eosin (H&E) to detect inflammatory infiltrates and myelin loss. Images were captured under a high-resolution Olympus DP71 microscope.

### Mast cells adoptive transfer

*Kit*^*W*^*/Kit*^*W−v*^ mice were reconstituted i.p. with WT BMDMCs generated from C57BL/6 as described above. Each mouse received 2 × 10^6^ cells in PBS.

### Histology

For histology, spinal cords were removed and immediately fixed in 10% neutral buffered formalin (Bio-optica) for 24 h. Tissues were then dehydrated, embedded in paraffin, sectioned into 3–4 μm slices and stained with hematoxylin and eosin stain. All images were visualized using a BX51 Olympus equipped with a high-resolution DP71 camera (Olympus) with a × 4, × 20 and × 40 objective with the analySIS image processing software (Olympus) or EVOS® FL Color Imaging System with a × 40 objective.

### Transmission electron microscopy (TEM)

Myelin loss investigation was assessed by TEM. Cerebellum sections were fixed with 1 ml 2% (w/v) paraformaldehyde and 3% glutaraldehyde in cacodylate buffer 0.1 M (pH 7.4), and then dehydrated with increasing concentrations of ethyl alcohol and propylene oxide. Samples were then embedded into acrylic resin, fixed and post-stained with 2% uranyl acetate. Micrographs were generated using a transmission electron microscope (EM 208, Philips).

### Flow cytometry

Flow cytometry was performed on a BD LSRFortessa™ cell analyzer (Becton Dickinson) and the data were analyzed using FlowJo software (Tree Star).

### Murine gut organoid culture

Murine gut organoid cultures were performed using intestinal crypts from the ileum of C57BL/6 mice. Crypts were isolated from murine small intestine by incubating for 30 min in Cell Recovery Solution (Corning) on ice. Isolated crypts were washed and mixed to Matrigel (Corning) and plated in 24 well plates. After polymerization of matrigel, 500 µl Intesticult Organoid Growth Medium (Stemcell) was added. The entire medium was changed every 2 days. For passage, organoids were removed from the matrigel and dissociated into single-crypt domains. On day + 14, cultures were stimulated with 3-IAld or DMSO 0.1%.

### β-hexosaminidase release assay

Degranulation was measured by the percentage of β-hexosaminidase released by IgE pre-sensitized BMDMCs challenged with DNP-BSA (Thermo Scientific; 1 µg/mL). 3-IAld (1 µM) or DMSO 0.1%, as vehicle control, were added overnight before Ag addiction. The samples were placed on ice and immediately centrifuged to pellet the cells. The enzymatic activity of β-hexosaminidase in supernatants and in the cell pellets was measured with p-nitrophenyl *N*-acetyl-β-d-glucosaminide (pNAG, Sigma-Aldrich; 0.34 mg/ml in sodium cytrate 0.1 M, pH 4.5) for 60 min at 37 °C. The reaction was stopped by adding sodium bicarbonate (0.1 M, pH 10.7). Product release was detected by absorbance at 405 nm. The extent of degranulation was calculated as the percentage of pNAG absorbance in the supernatants over the sum of absorbance in the supernatants and in cell pellets solubilized in detergent.

### Western blotting for AhR translocation

For each condition, 8 × 10^6^ BMDMCs were seeded in a 6-well-plate in RPMI medium and treated with 3-IAld or 3-IAld and DNP for 1 h. 3-IAld and DNP final concentrations were respectively 10 µM and 100 ng/ml. Vehicle-treated BMDMCs were used as control. BMDMCs treated with 3-IAld and DNP were pre-stimulated with IgE overnight in IL-3 enriched RPMI medium and washed in PBS prior 3-IAld and DNP incubation.

Nuclear and cytoplasmic extracts were obtained with Nuclear Extract Kit (Active Motif) in according to manufacturing’s instructions. An amount of 40 μg and 20 μg of protein extracts were used for nuclear and cytosolic lysates, respectively. Samples were loaded on 8% SDS-PAGE gels and the electrophoresis were performed in Tris–Glycine buffer at 110 V for approximately 90 min. The blotting was carried out on a Nitrocellulose membrane (BioRad), in Transfer Tris–Glycine buffer (20% methanol) at 300 mA for three hours in a cold room at 4 °C. The membrane was then blocked in 5% Bovine Serum Albumin TBS-T (Tris-buffered saline, 0.05% Tween-20) for 1 h.

Primary antibodies were incubated overnight at 4 °C. Three washes 10 min each were performed with TBST before the addition of secondary antibody for two hours at room temperature. Three additional washes were performed as previously described, before imaging on Odyssey CLx (LI-COR Biosciences). Protein detection was performed using Image Studio Ver 5.2.

Primary antibodies: goat anti-mouse AhR (Santa Cruz sc-8089), mouse anti-actin (BD Biosciences, 612,657) and rabbit anti-laminin (ThermoFisher).

Secondary antibodies: donkey anti-goat CF770 (SAB 4,600,374), goat anti-mouse CF770 (SAB4600214), goat anti-mouse CF680 (SAB4600361) and goat anti-rabbit (SAB4600215); all from SIGMA-Aldrich.

### Patients and study approval

Serum samples were collected from 35 consecutive multiple sclerosis (MS) patients in the outpatient MS Center at S. Maria della Misericordia Hospital, University of Perugia, Perugia (IT) over a period of 6 months (from June 2017 to November 2017). Patient characteristics are summarized in Table S1. Briefly, mean age at serum sampling was 38.9 ± 10.6 years and the female to male ratio was 2.5. Mean disease duration (i.e. the time between the first clinical manifestation of the disease and serum sampling) was 11.6 ± 19.5 years. Out of the 35 patients, 29 (82.9%) had relapsing remitting MS and six (17.1%) had either primary or secondary progressive MS. At the time of serum sampling, most patients (68.6%) were under disease modifying therapies (DMTs). In addition, most patients did not have evidence of disease activity in the 30 days preceding serum sampling: only 11.4% had a recent relapse, 17.1% had new lesions on brain magnetic resonance imaging (MRI) T2-weighted sequences and 5.7% had gadolinium enhancing lesions in a recent brain MRI scan. The mean neurological disability, as assessed by means of the Expanded Disability Status Scale (EDSS), was 2.1 ± 1.5. Clinical and neuro-radiological characteristics were recorded at the time of serum sampling.

All the experiments were performed in accordance with relevant guidelines and regulations. The regional CEAS Ethics Committee from Umbria Regione (Italy) approved the study (CEAS n. 2925/16). All methods were carried out in accordance with relevant guidelines and regulations. All experimental protocols were approved by CEAS Ethics Committee. Informed consent was obtained from all subjects and/or their legal guardian(s).

### Statistical analyses

The normal distribution of human serum 3-IAld was verified by Shapiro–Wilk test. The differences between serum 3-IAld concentrations in different groups were assessed by Mann–Whitney test. Correlation between continuous variables was assessed by Spearman’s rank correlation test. All human tests were two-sided and statistical significance was set at *p* < 0.05. Statistical analyses were performed in Graphpad Prism 9, version 9.5.1. Murine data are expressed as the means ± SD. Horizontal bars indicate the means. For multiple comparisons, p values were calculated by One-way ANOVA or Two-way ANOVA with Bonferroni’s post hoc test. For single comparisons, *p* values were calculated by two-tailed Student’s t test. The data reported are from one representative experiment out of three to five independent experiments.

### Supplementary Information

Supplementary Video 1.

Supplementary Information 1.

## Data Availability

The datasets generated and/or analysed during the current study are available from the corresponding author on reasonable request.
