# Supplementary material for: A microbially produced AhR ligand promotes a Tph1-driven tolerogenic program in multiple sclerosis
Source: Sci Rep. 2024 Mar 20;14:6651. doi: 10.1038/s41598-024-57400-8 (PMC10954611; doi:10.1038/s41598-024-57400-8)
Supplement: Supplementary file 2 — Supplementary Information 1. [file 41598_2024_57400_MOESM2_ESM.docx]

**
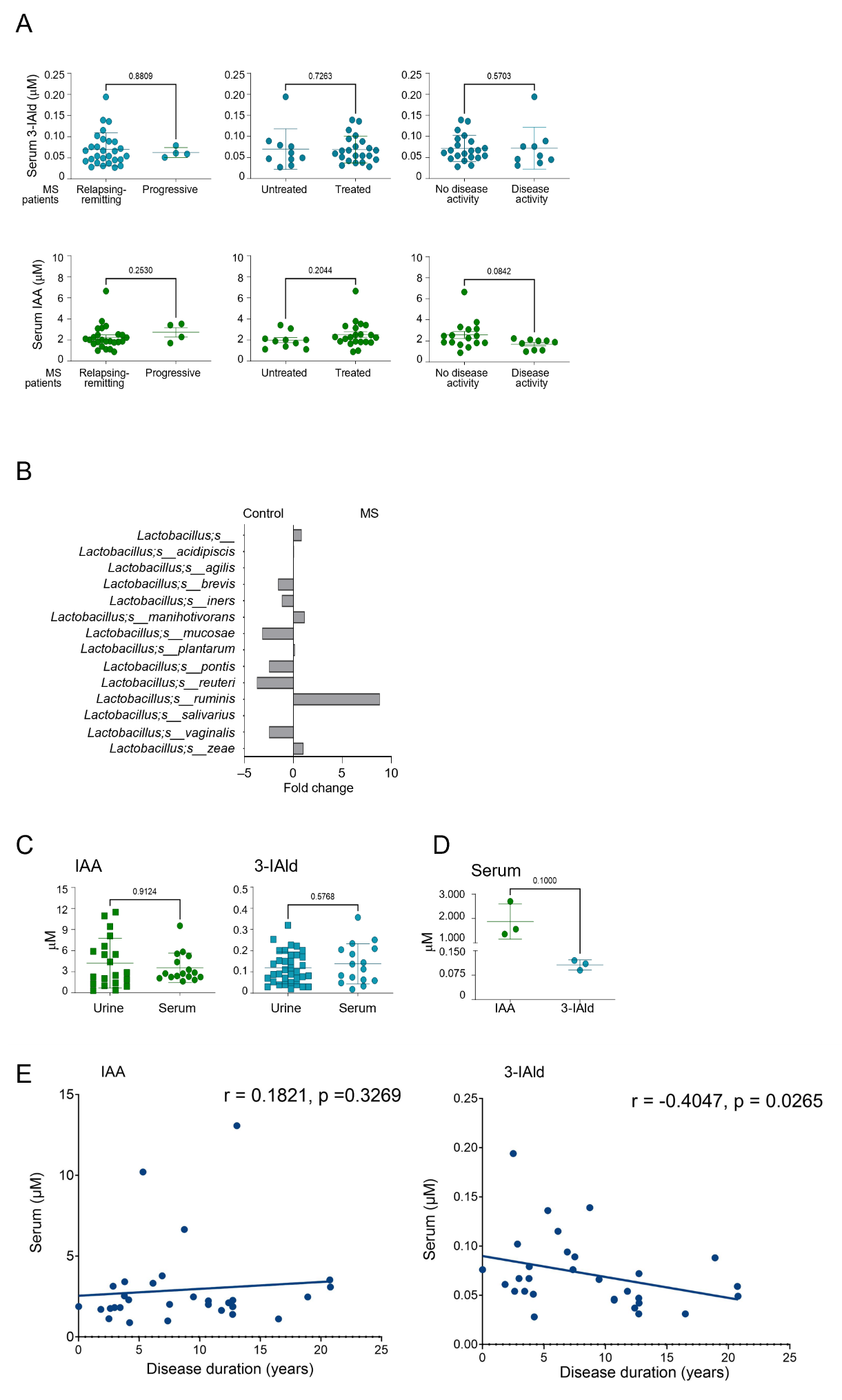
**

**Figure S1. IPyA metabolites detection in biological fluids**

**(A**) IAA and 3-IAld serum levels in MS patients. (**B**) Abundance of *Arat*^+^ bacteria in MS patients from a previously published metagenomics dataset that included subjects with MS (MS, n = 60) and healthy controls (*n* = 43) (Jangi et al., 2016), we identified 14 bacterial strains *Arat*+ (36%). All statistical analyses were made with the R software. Raw counts were quantile normalized with metagenome Seq R package and differential abundance of species was evaluated. Data in C shows the log2 (FoldChange) between patients and controls. Differential abundance is not significant within this dataset. (**C**) IAA and 3-IAld levels in urine and serum of healthy subjects. (**D**) IAA and 3-IAld levels in serum of WT naïve mice. (**E**) Correlation of 3-IAld and IAA serum levels with disease duration in MS patients. The normal distribution was verified by Shapiro-Wilk test and correlation was assessed by Spearman’s rank correlation test. Tests were two-sided and significance was set at p< 0.05.Statistical analysis was performed using a Two-tailed Student’s t test, nonparametric Mann-Whitney U test (A, C, D). Data represent the means ± SD from three independent experiments; ∗p < 0.05.

**
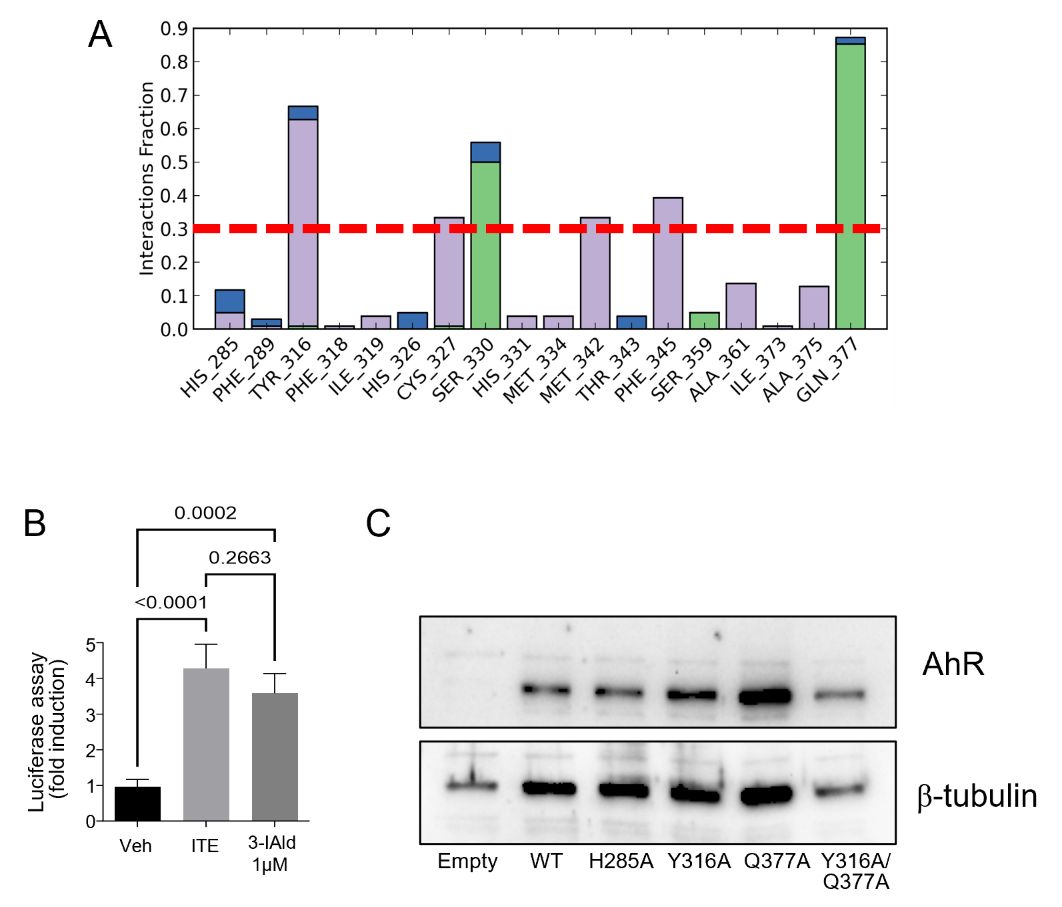
**

**Figure S2. Characterization of the 3-IAld – AhR binding model**

(**A**) MD simulation interaction plot reporting the occupancy (y-axis; interactions fraction) of intermolecular hydrogen bonds (green bars), aromatic/hydrophobic contacts (purple bars) and polar interactions (blue bars) for AhR binding site residues (x axis) engaged by 3-IAld along the trajectory. A cut-off value of 30% (red dashed line) was used to select the most relevant interactions and key binding site residues. (**B**) Transactivation activity of human AhR by 3-IAld or ITE in transfected murine embryonic fibroblasts (MEFs). Data represent the means ± SD from three independent experiments. (**C**) AhR-deficient MEFs were transfected with WT or mutant AhR (Q377A, H285A, Y316A, and Y316A/Q377A). After 24 h, cells were incubated with cycloheximide (CXM) (10 µg ml^−1^) and harvested at different times, lysed, and analysed for AhR expression by immunoblotting, using a specific antibody. β-tubulin was used as a loading control. Data are representative of three experiments. Statistical analysis was performed using a One-way ANOVA, Bonferroni post hoc test. Data represent the means ± SD from three independent experiments; ∗∗∗p < 0.001. ∗∗∗∗p < 0.0001.

**
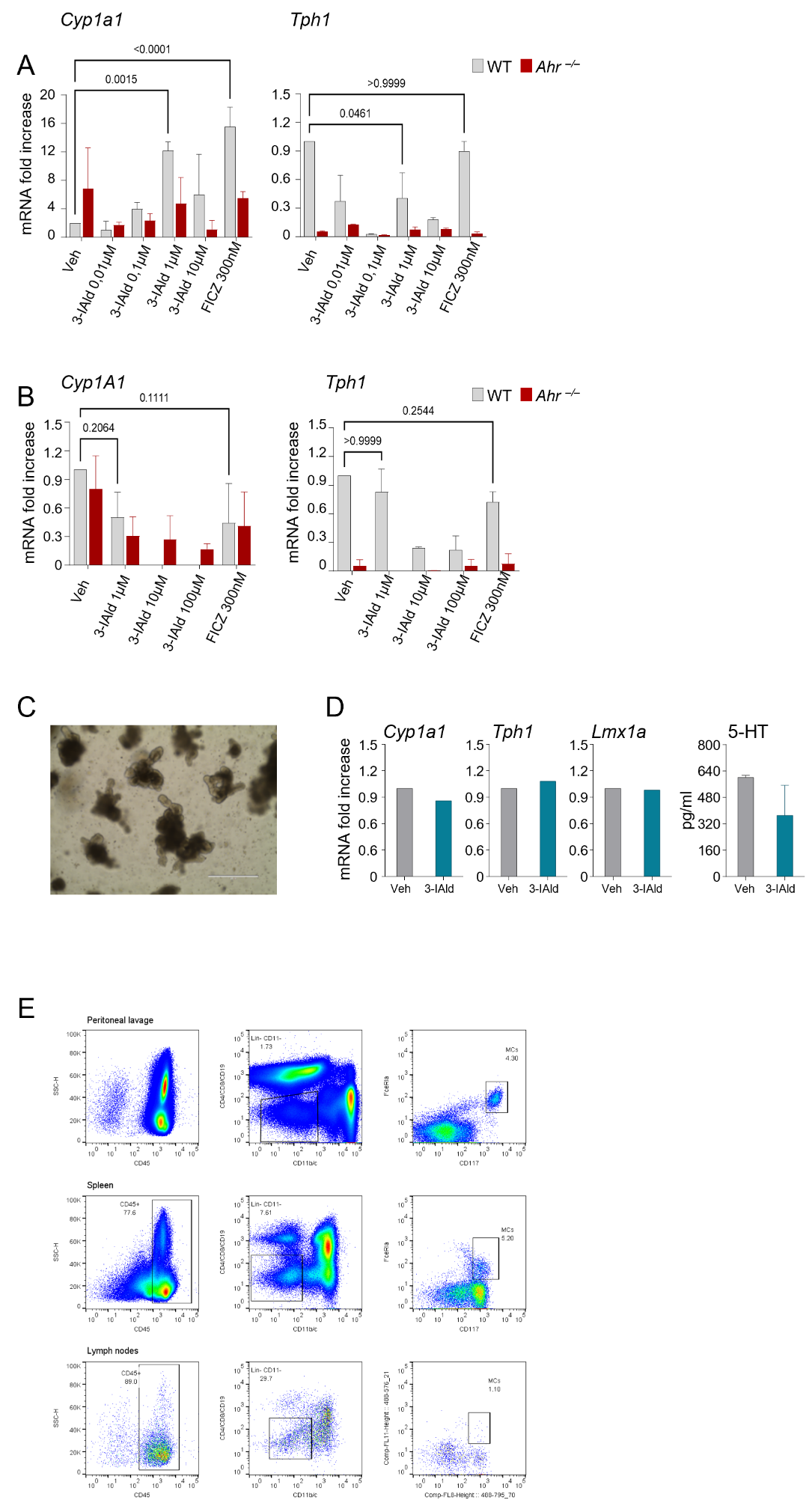
**

**Figure S3. 3-IAld activates Tph1 in mast cells**

*Cyp1a1* and *Tph1* mRNA expression in (**A**) WT and *Ahr*^−/−^ CD4^+^ T cells and (**B**) on non-IgE sensitized BMDMCs treated with 3-IAld or vehicle (Veh-DMSO 0.1% of culture medium) overnight. (**C**) WT gut organoids on day 14 of culture. Scale bar = 400 µm (**D**) Fold increase in relative gene expression and 5-HT detection in WT 3-IAld-(1 µM) or vehicle (DMSO 0.1%) treated gut organoids taken on day 14 of culture. (**E**) Representative gating strategy for murine mast cell sorting.

Statistical analysis was performed using a Two-way ANOVA, Bonferroni post hoc test. Data represent the means ± SD from three independent experiments; ∗∗p < 0.01, ∗∗∗∗ p< 0.0001.

**
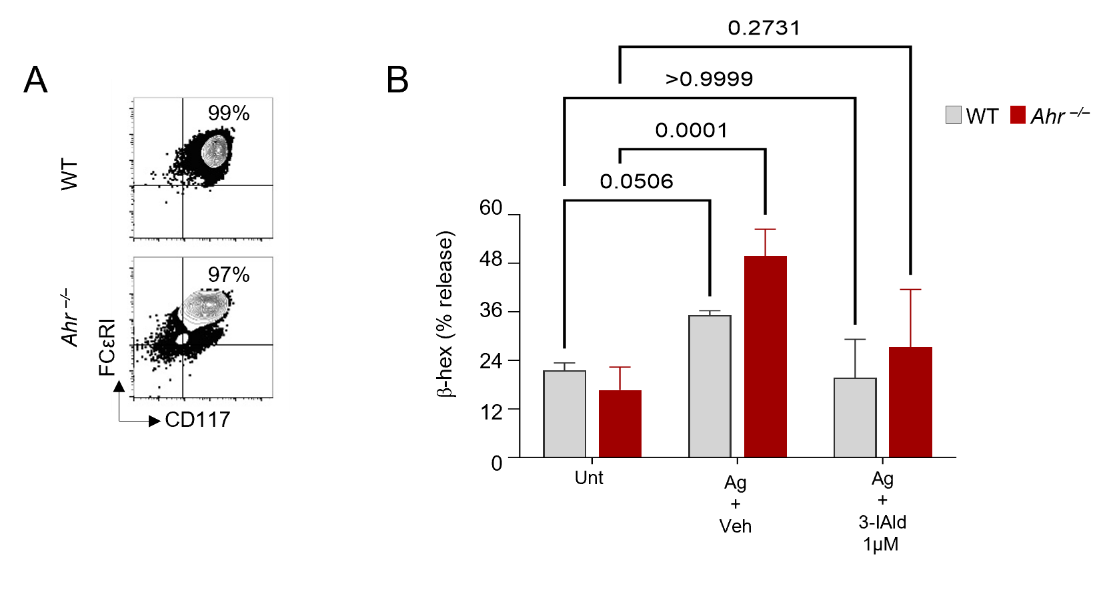
**

**Figure S4. 3-IAld does not affect mast cell degranulation**

Mast cells were derived from bone marrow precursors by culturing stem cells of 4-week-old female WT and *Ahr^−/−^* mice in RPMI 1640 supplemented medium. (**A**) Purity was measured by FACS by monitoring FcεRI, cKit receptor (CD117) expression. (**B**) Quantification of β-hexosaminidase release in WT and *Ahr*^−/−^ IgE-activated BMDMCs. 3-IAld (1 µM) or Veh, as vehicle control (DMSO 0,1%), were added overnight to IgE pre-sensitized BMDMCs that were challenged with Ag (DNP-BSA) the day after. Statistical analysis was performed using a Two-way ANOVA, Bonferroni post hoc test. Data represent the means ± SD from three independent experiments; ∗p < 0.05, ∗∗p < 0.01,  *n.s.*, not significant.

**
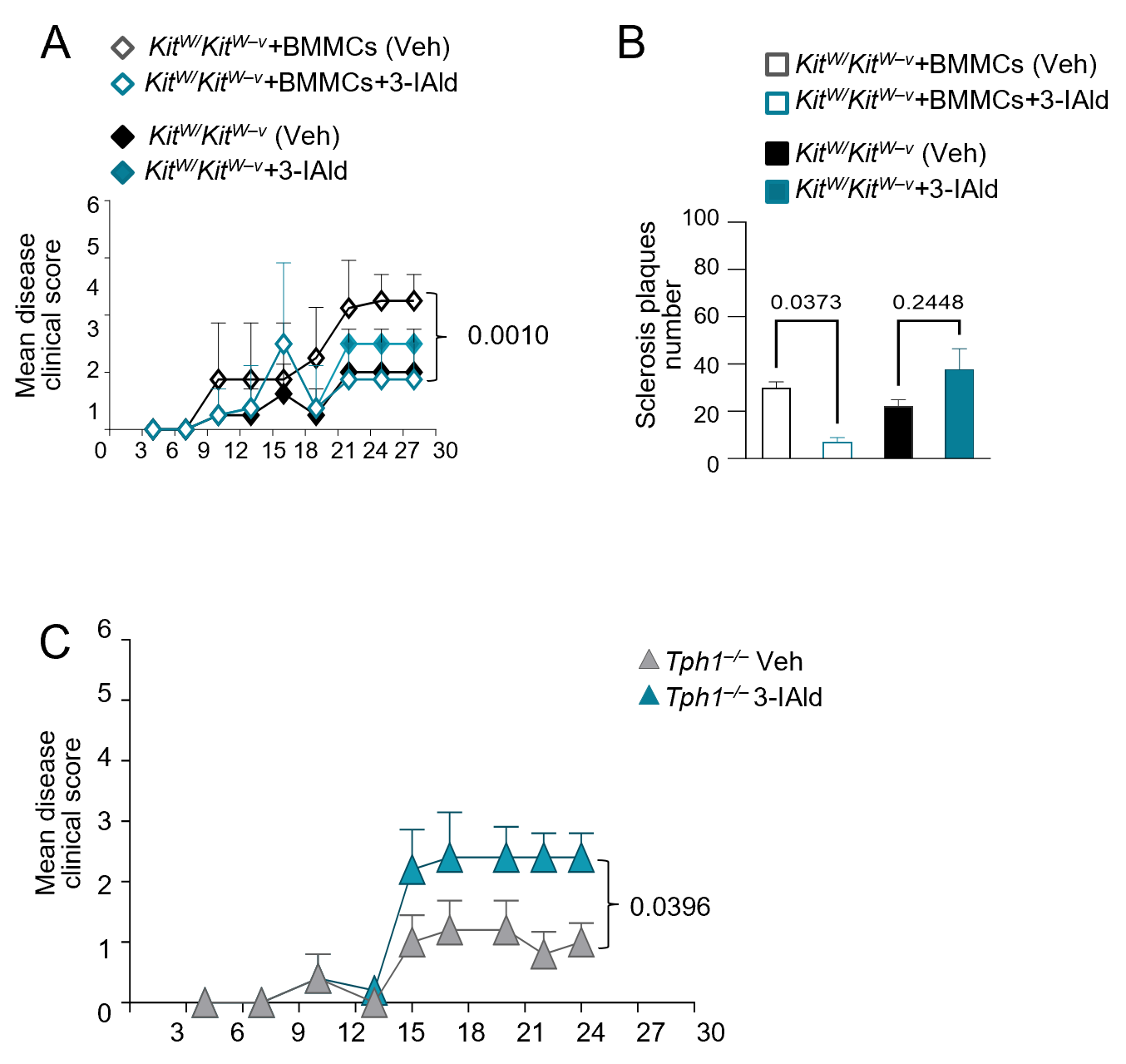
**

**Figure S5. MCs reconstitution restores 3-IAld protection in *Kit^W^/Kit^W–v^* mice**

EAE was induced in *Kit^W^/Kit^W–v^* reconstituted or not reconstituted mice with WT BMDMCs. 3-IAld (0.36 mg/mouse in olive oil) or Veh (DMSO 0.1% of olive oil) were injected i.p. as indicated in Figure 4A. (**A**) Assays were performed at 30 dpi and data were pooled from two independent experiments (*n* = 4 mice per group). Statistical analysis was performed using a Multiple T test. (**B**) Quantification of sclerosis plaques. **(C)** Quantification of the mean clinical score in a mouse model of EAE following systemic treatment of 3-IAld. Statistical analysis was performed using a One-way ANOVA, Bonferroni post hoc test. Data represent the means ± SD from two independent experiments; ∗p < 0.05. *n.s.*, not significant.

**
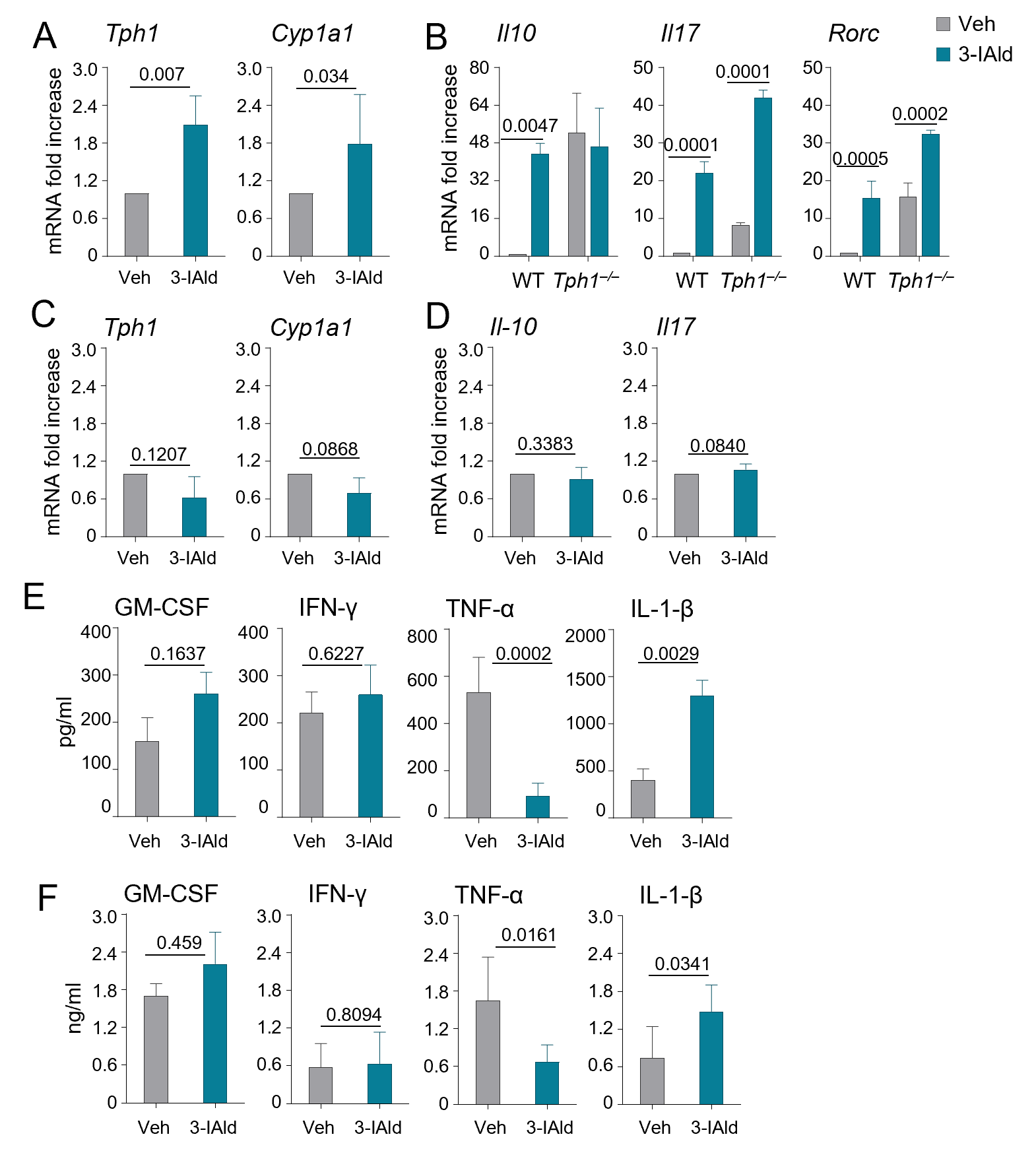
Figure S6. 3-IAld affects systemic inflammation in spinal cord and cervical lymph nodes**

EAE was induced in WT mice, 3-IAld (0.36 mg/mouse in olive oil) or Veh (DMSO 0.1% of olive oil) were injected i.p. (**A, B, F**) Fold increase in relative gene expression (**A, B**) in cervical lymph nodes and cytokine measure via ELISA (**F**). (**C, D, E**) Fold increase in relative gene expression (**C, D**) in spinal cord and cytokine measure via ELISA (**E**). Statistical analysis was performed using Two-tailed Student’s t test, nonparametric Mann-Whitney U test (A, C, D, E, F); Two-way ANOVA, Bonferroni post hoc test (B). ∗p < 0.05, ∗∗p < 0.01, ∗∗∗ p< 0.001, ∗∗∗∗ p< 0.0001.

**SUPPLEMENTAL TABLES**

**Table. S1. Patients characteristics**

Continuous variables are reported as the means ± SD. Categorical variables are reported as a number and percentage. Legend: DMT: disease modifying therapy. EDSS: expanded disability status scale. Gd+: gadolinium enhancing. MRI: magnetic resonance imaging. PMS: progressive multiple sclerosis (either primary or secondary progressive multiple sclerosis). RRMS: relapsing remitting multiple sclerosis.


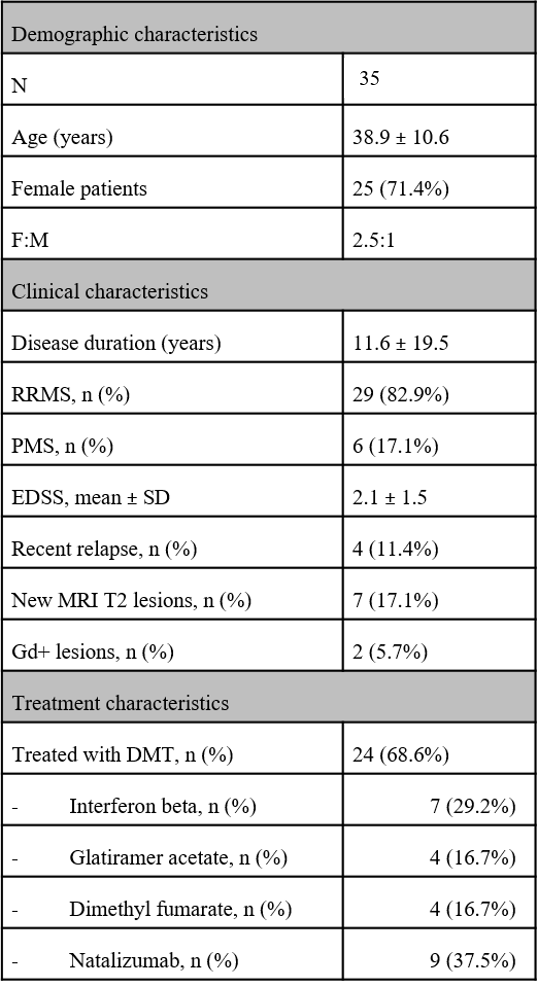


**Table S2:** List of chromatographic retention time (RT), selected MRM parameters, declustering potential (DP), entrance potential (EP), collision energy (CE), cell exit potential (CXP) for each measured analyte.

| Q1  (m/z) | Q3  (m/z) | RT  (min) | analyte | DP | EP | CE | CXP |
| --- | --- | --- | --- | --- | --- | --- | --- |
| 183.1 | 136 | 10.8 | indol-3-acetic-acid D7 | 36 | 10 | 22 | 9 |
| 183.1 | 109 | 10.8 | indol-3-acetic-acid D7 | 36 | 10 | 43 | 6 |
| 176.1 | 130 | 10.9 | indol-3-acetic-acid | 36 | 10 | 22 | 9 |
| 176.1 | 103 | 10.9 | indol-3-acetic-acid | 36 | 10 | 43 | 6 |
| 146.1 | 118 | 10.7 | indole-3-carboxy-aldehyde | 48 | 10 | 25 | 7 |
| 146.1 | 91 | 10.7 | indole-3-carboxy-aldehyde | 48 | 10 | 28 | 7 |

**Table S3:** Chromatographic conditions.

| Time (min) | Flow (mL/min) | B (%) |
| --- | --- | --- |
| 0 | 0.300 | 0 |
| 11 | 0.300 | 40 |
| 13 | 0.300 | 95 |
| 21 | 0.300 | 95 |
| 22 | 0.300 | 0 |
| 33 | 0.300 | 0 |

**REFERENCES**

Jangi, S., Gandhi, R., Cox, L.M., Li, N., von Glehn, F., Yan, R., Patel, B., Mazzola, M.A., Liu, S., Glanz, B.L.*, et al.* (2016). Alterations of the human gut microbiome in multiple sclerosis. Nat Commun *7*, 12015.
